# Sodium Intake and Disease: Another Relationship to Consider

**DOI:** 10.3390/nu15030535

**Published:** 2023-01-19

**Authors:** Caitlin Baumer-Harrison, Joseph M. Breza, Colin Sumners, Eric G. Krause, Annette D. de Kloet

**Affiliations:** 1 Department of Physiology and Aging, College of Medicine, University of Florida, Gainesville, FL 32603, USA; 2 Center for Integrative Cardiovascular and Metabolic Disease, University of Florida, Gainesville, FL 32610, USA; 3 Center for Smell and Taste, University of Florida, Gainesville, FL 32610, USA; 4 Evelyn F. and William L. McKnight Brain Institute, University of Florida, Gainesville, FL 32610, USA; 5 Department of Psychology, College of Arts and Sciences, Eastern Michigan University, Ypsilanti, MI 48197, USA; 6 Department of Pharmacodynamics, College of Pharmacy, University of Florida, Gainesville, FL 32610, USA

**Keywords:** sodium appetite, taste, blood pressure, stress, cardiometabolic disease

## Abstract

Sodium (Na^+^) is crucial for numerous homeostatic processes in the body and, consequentially, its levels are tightly regulated by multiple organ systems. Sodium is acquired from the diet, commonly in the form of NaCl (table salt), and substances that contain sodium taste salty and are innately palatable at concentrations that are advantageous to physiological homeostasis. The importance of sodium homeostasis is reflected by sodium appetite, an “all-hands-on-deck” response involving the brain, multiple peripheral organ systems, and endocrine factors, to increase sodium intake and replenish sodium levels in times of depletion. Visceral sensory information and endocrine signals are integrated by the brain to regulate sodium intake. Dysregulation of the systems involved can lead to sodium overconsumption, which numerous studies have considered causal for the development of diseases, such as hypertension. The purpose here is to consider the inverse—how disease impacts sodium intake, with a focus on stress-related and cardiometabolic diseases. Our proposition is that such diseases contribute to an increase in sodium intake, potentially eliciting a vicious cycle toward disease exacerbation. First, we describe the mechanism(s) that regulate each of these processes independently. Then, we highlight the points of overlap and integration of these processes. We propose that the analogous neural circuitry involved in regulating sodium intake and blood pressure, at least in part, underlies the reciprocal relationship between neural control of these functions. Finally, we conclude with a discussion on how stress-related and cardiometabolic diseases influence these circuitries to alter the consumption of sodium.

## 1. Introduction

Sodium (Na^+^), most abundantly consumed in the form of table salt (NaCl), is essential for survival as it plays a pivotal role in numerous physiological processes, such as muscle and nerve function, and the maintenance of body fluid and blood pressure homeostasis. Changes in plasma sodium concentration, be they elevations or reductions, can negatively impact health and therefore the intake of sodium is tightly regulated by orchestration of the gustatory, neural, endocrine, cardiovascular, and renal systems. Along these lines, terrestrial mammals have evolved physiological adaptations, such as the renin–angiotensin–aldosterone system (RAAS) and sodium appetite that are fundamental to maintaining sodium levels in times of deficit and other mechanisms, such as oxytocin, that maintain these levels during surplus. An additional, often overlooked component to sodium balance is one’s ability to taste salt (NaCl). The sensitivity of salt taste undergoes plasticity based on physiological condition and during sodium depletion, for example, there is a hedonic shift that leads to enhanced palatability of higher, normally aversive, concentrations of NaCl [1,2]. This and the other adaptations that occur during sodium deficit drive the consumption of higher concentrations and quantities of sodium to ultimately restore homeostasis.

While the consumption of adequate sodium is critically important to maintaining health, and its underconsumption clearly causes physiological ailments, its overconsumption is perhaps more widely-acknowledged as problematic. Sodium is easily attainable within the human diet and we have developed a sodium preference and consequent tendency to consume sodium even when we are replete. This leads to the frequent consumption of sodium beyond physiological need, the adverse health implications of which are generally accepted. For example, excess sodium intake can impact the body’s responses to physiological and psychological stressors and its level is, in turn, positively correlated with blood pressure in animals and humans [3,4,5,6]. On the other hand, the inverse relationship—how stress-related and cardiometabolic pathophysiology impacts sodium intake—is elusive.

**Figure 1:**
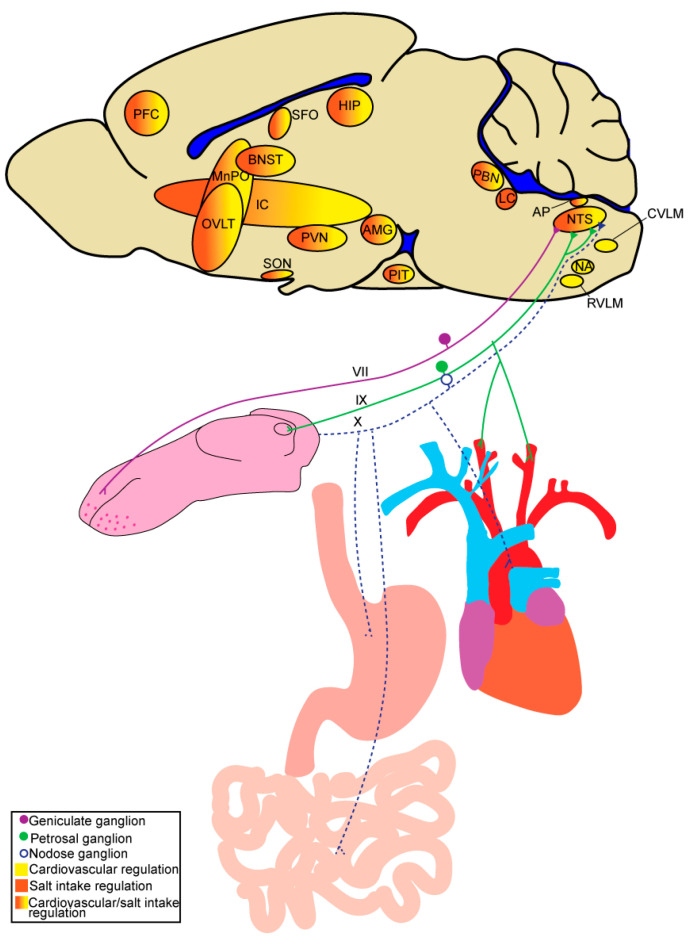
Schematic highlighting peripheral inputs and brain regions involved in salt intake and/or cardiovascular regulation. AMG, amygdala; AP, area postrema; BNST, bed nucleus of the stria terminalis; CVLM, caudal ventrolateral medulla; HIP, hippocampus; IC, insular cortex; LC, locus coeruleus; MnPO, median preoptic nucleus; NA, nucleus ambiguous; NTS, nucleus of the solitary tract; OVLT, organum vasculosum of the lamina terminalis; PBN, parabrachial nucleus; PFC, prefrontal cortex; PIT, pituitary; PVN, paraventricular nucleus of the hypothalamus; RVLM, rostral ventrolateral medulla; SFO, subfornical organ; SON, supraoptic nucleus.

In this review, we consider the impact of stress and cardiometabolic diseases on sodium intake and blood-pressure regulation. There are numerous points of overlap between the neural circuitries (Figure 1) and endocrine factors that regulate sodium intake and those that regulate blood pressure. First, we describe the mechanism(s) that regulate each of these processes independently. Then, we highlight the points of overlap and integration of these processes. We conclude with a discussion on how stress and cardiometabolic diseases dysregulate these mechanism(s) and thereby increase both salt intake and blood pressure.

## 2. The Drive to Consume Sodium and Salt Taste

Sodium is the most abundant electrolyte in extracellular fluid and, as such, plasma volume and osmolality are dictated primarily by sodium concentration, the level of which is maintained by balancing sodium intake with excretion. Changes in sodium balance can be detrimental to physiology as it is necessary for numerous processes, such as neural function, muscle contraction, and metabolism.

### 2.1. Sodium Appetite

Chronic sodium depletion leads to insufficient levels of sodium in the body (hyponatremia) resulting in dehydration of extracellular fluid and a decrease in total fluid volume (hypovolemia) and osmolality. The challenge of consuming adequate sodium from one’s diet is faced by many terrestrial animals who, as a result, have evolved physiological adaptations to conserve and replete sodium levels under conditions of depletion. One such adaptation is sodium appetite, which increases the drive to consume high concentrations of sodium to reestablish homeostasis when depleted. Sodium levels are detected by the brain and various peripheral organ systems, including the heart, vasculature, and kidneys, that then regulate sodium intake accordingly. In a sodium-deplete state, signals from peripheral organ systems, endocrine factors, and osmo-sensing brain regions work collectively to stimulate sodium appetite and restore sodium to optimal levels for survival. Humans also have a physiological need to maintain sodium balance, however industrialization has made sodium easily attainable in the diet in the form of NaCl, or table salt, and because of this, we rarely experience sodium appetite. Instead, humans in industrialized countries have developed a salt preference in which we consume NaCl in amounts exceeding what is physiologically necessary due to its high palatability.

### 2.2. Salt Preference and Taste

Salt preference is the drive to consume salt, in the form of NaCl, due to its high palatability regardless of physiological need for sodium. Industrialization has further exacerbated this preference as salt (NaCl) is added to various processed foods for palatability, preservation, and processing purposes. Adults worldwide consume sodium in excess—at levels nearly double the amount of what is recommended [7]. The negative health implications of overconsuming sodium are well-documented and efforts to reduce sodium intake to prevent or treat conditions related to sodium intake, such as hypertension, have been largely unsuccessful [3,8,9,10]. Understanding salt preference can help guide interventions aimed at reducing sodium intake. Salt taste, described in more detail in the following section, is a critical driver of excess sodium intake as an individual’s preference will dictate how pleasurable they find salt. fMRI studies revealed individual daily salt intake and salt preference are significantly correlated with increased neural activity in gustatory processing areas including the insular cortex, orbitofrontal cortex (OFC), and parahippocampus [11,12].

It has been indicated that one’s diet can influence their taste responses and preferences [13]. While salt preference is likely shaped by innate components due to physiological need for sodium, it is also acquired and can be impacted by dietary sodium exposure throughout the lifespan. Rats exposed to high NaCl in prenatal and early postnatal development exhibit an increased preference for NaCl in adulthood [14,15,16,17]. A similar relationship is observed in humans regarding early dietary experience and sodium preference. Infants exposed to foods higher in sodium displayed more acceptance for salty foods at six months and preschool age [18,19]. It has been proposed that such increases in sodium preference that result from early dietary NaCl exposure may be due to changes in salt taste processing or changes in systems regulating sodium balance [20]. These studies are suggestive of a direct link between dietary sodium and salt taste preference; a relationship that is maintained in adulthood. Along these same lines, adults asked to supplement sodium in their diet preferred higher concentrations of NaCl in soup, with this increased preference lasting weeks after cessation of salt supplementation in the diet [21]. The implication is that excess sodium consumption increases preference for salty foods and further exacerbates sodium overconsumption. Due to this direct relationship, it is reasonable to expect that reducing dietary sodium would decrease preference for salty foods. Healthy adults placed on long-term (5 months) low sodium diet preferred lower concentrations of salt in food compared to their pre-diet preferences [22]. While the underlying mechanisms of our salt preference remain debatable, there is no question that humans overconsume salt and this is largely dependent on the palatability of salt.

The sense of taste is critical in identifying nutrients and toxins upon ingestion of substances from the environment and plays a pivotal role in diet selection and health. Sweet, umami, and salty tastes signal the presence of nutrients and are inherently appetitive, while sour and bitter tastes signal the presence of potential toxins and are aversive. Further highlighting the physiological importance of sodium, is the dedication of entire taste modality to detecting it. Salt taste is not only necessary for the detection of sodium, but it is also important in the regulation of sodium homeostasis. Over the last 20 years, the transduction mechanisms behind sweet, bitter, sour, and umami tastes have become more understood, while the mechanisms of salt taste remain enigmatic.

A breakthrough in our understanding of salt taste occurred in the 1980s, when DeSimone and colleagues found that amiloride, a diuretic known to block epithelial sodium channels (ENaCs) in other tissues, attenuated chorda tympani responses to lingual NaCl but not potassium chloride (KCl) stimulation in rats [23,24]. This led them to deduce the taste bud must include an amiloride-sensitive sodium transport pathway for salt taste. Shortly after identifying the sodium selective amiloride-sensitive salt-taste transduction pathway, another pathway for salt-taste transduction was identified. Whole nerve chorda tympani recordings of NaCl taste responses in rats revealed nerve fibers fall in to two groups, one being sodium selective and amiloride-sensitive and the second being cation non-selective and amiloride-insensitive [25]. Although some evidence has suggested the involvement of a variant of the TRP channel family for the amiloride-insensitive pathway, further studies examining neural and behavioral responses in TRPV1 knockout mice [26,27] and neural responses in the presence of TRPV1 antagonists in rats [28] have been contradictory.

These distinct gustatory afferent pathways for salt taste are not only characterized by their amiloride-sensitivity but also by the concentration of NaCl that recruits each pathway. The amiloride-sensitive pathway responds to low concentrations of sodium (at or below isotonic) and is involved in appetitive behavioral responses to NaCl [29]. While the amiloride-insensitive pathway responds to high concentrations of sodium and is involved in aversive behavioral responses to NaCl [30,31]. In the sodium-depleted state, however, animals show strong preferences for hypertonic (0.3–0.5 M) NaCl and amiloride-sensitive neurons are necessary for sodium appetite [32]. Thus, while the amiloride-sensitive pathway is involved in appetitive behavioral responses to low concentrations, sodium depletion results in decreased spike rates in amiloride-sensitive neurons, which might influence the acceptance for normally aversive hypertonic NaCl solutions. This appetitive-aversive balance by the gustatory system in salt taste is reflective of the need to maintain sodium balance and shows the importance of the gustatory system in regulating sodium intake.

There are three types of morphologically and physiologically distinct taste receptor cells comprising taste buds. Type III cells are involved in sour taste, type II cells are involved in sweet and umami taste, and type I cells, whose role in taste transduction is debated, are suggested to be involved in salt taste [33,34]. From the taste bud, gustatory signals are transmitted to the brain through vagal, glossopharyngeal, and facial nerve innervation of the pharynx, posterior tongue, and anterior tongue, respectively. However, the majority of gustatory signals are transmitted through the glossopharyngeal nerve and two branches of the facial nerve, the chorda tympani and greater superficial petrosal. While all three nerves respond to salt, the chorda tympani is most responsive and critical for normal salt taste function [32,35,36,37,38]. The central neural circuitry that is involved in the sensation and perception of salt taste has been reviewed elsewhere [39,40,41] and share many points of overlap with those mediating sodium appetite and cardiovascular homeostasis (discussed below) (Figure 1).

## 3. Neural and Hormonal Control of Sodium Intake

### 3.1. Neural Circuitry Mediating Sodium Appetite

The brain receives and integrates signals pertaining directly to plasma osmolality, as well as endocrine and neural input arising from peripheral organ systems and responds to these collective stimuli by spurring or suppressing sodium appetite. The neural circuitry that mediates these processes is multifaceted and has been described in detail in several comprehensive reviews [42,43,44,45]. Key to this circuitry, is the ability to detect changes in the internal milieu, which is accomplished, in part, by sensory circumventricular organs (CVOs) around the third and fourth ventricle, including the subfornical organ (SFO), organum vasculosum of the lamina terminalis (OVLT), and area postrema (AP) [46,47]. These areas receive and integrate signals from the bloodstream and are highly interconnected with other brain regions that are also involved in sodium appetite, such as the nucleus of the solitary tract (NTS), parabrachial nucleus (PBN), locus coeruleus (LC), paraventricular nucleus of the hypothalamus (PVN), supraoptic nucleus (SON), and bed nucleus of the stria terminalis (BNST) [48,49,50,51,52,53] (Figure 1). In support of roles for the CVOs in sodium appetite, threats to sodium balance differentially activate the CVOs, and their lesion profoundly influences salt intake. For example, SFO lesion decreases salt intake in sodium-deplete rats, induced by acute treatment of furosemide [54,55], while lesion of the AP increases salt intake in replete rats [56].

Sodium appetite is controlled, at least in part, by the detection of changes in sodium concentration ([Na^+^]) by osmosensitive neurons within the brain [57,58,59,60]. These neurons are located within the SFO, OVLT, median preoptic nucleus (MnPO), PVN, and SON [59,60,61,62,63,64]. Osmosentive neurons express various [Na^+^]-sensitive channels, such as TPRV1 (*N*-terminal variant of “transient receptor potential vanilloid 1”), TRPV4 (“transient receptor potential vanilloid 4”), and Na_x_ (sodium sensor channel). Studies support expression of all three [Na^+^]-sensitive channels in neurons of the SFO and OVLT [60], expression of TRPV1 and TRPV4 in PVN and SON neurons [63,65,66], and Na_x_ in MnPO neurons involved in salt intake regulation [67]. In addition to direct osmosensation by the PVN and SON, they receive projections from the SFO and OVLT involved in sodium-appetite regulation [68]. The PVN and SON synthesize and secrete several neuropeptides (e.g., oxytocin) in response to changes to plasma osmolality and volume. As will be discussed below, such neuropeptides, in turn, also impact the neural circuitry that mediates sodium appetite.

Another key integrator of information from the periphery and regulator of sodium appetite and intake in response to changes in the internal milieu is the NTS. Vagal sensory afferents innervate visceral organs, including cardiovascular and gastrointestinal tissues to detect stretch and/or osmolality [69,70,71,72,73,74], and terminate in the NTS to provide the brain with information pertaining to the status of these peripheral systems. Lesion of the commissural nucleus of the NTS increases sodium intake in sodium-deplete rats [75], suggesting an inhibitory role of the area in sodium-appetite regulation; however, the role of the NTS is likely more complex, as the NTS is a highly heterogenous brain area containing excitatory and inhibitory neurons that differently regulate numerous vital functions. The NTS serves as the first central point of relay for gustatory and visceral sensory information, thus it is anatomically positioned to integrate these signals to regulate salt intake accordingly. Evidence supports communication between the caudal (visceral) NTS and rostral (gustatory) NTS [76], however the role these projections play in salt intake remains unknown. As such, disease-states that disrupt gustatory or visceral inputs into the NTS may alter the other and impact salt intake regulation.

The PBN, also located within the hindbrain, is involved in sodium-appetite regulation and receives input from the NTS [77] and AP [48]. Single-unit recordings from the PBN of sodium-deprived rats exhibit diminished neural responses to NaCl applied to the tongue [78], similar to other gustatory regions (e.g., NTS), suggesting that the PBN is involved in gustatory-mediated regulation of sodium appetite. The role of the PBN, more specifically the lateral PBN, in sodium appetite has been described in detail in [79]. Studies in rats have also implicated the pre-LC, an area located just rostral to the LC in the hindbrain that receives input from the AP, in sodium appetite [48]. Rats maintained on a low-sodium diet for over one week exhibited increased c-Fos expression induced by sodium depletion in neurons of the PBN and pre-LC [80]. In mice, neurons that are sensitive to sodium-depletion appear to be expressed in the rostral LC as opposed to the pre-LC [81]. As visceral sensory relay centers, the PBN and pre-LC send information pertaining to sodium balance to various forebrain regions involved in integrating signals to regulate salt intake. Neurons shown to be activated by sodium depletion in the PBN and pre-LC project to areas, such as the PVN, BNST, and thalamus in rats [82]. Projections from the PBN to central nucleus of the amygdala (CeA) have also been shown to play an important role in regulating sodium appetite in rats [83,84,85].

In summary, the brain senses and receives information pertaining to changes in the internal milieu. Changes in plasma osmolality are detected by osmosensitive neurons within CVOs, the PVN, and SON. The NTS receives information pertaining to blood pressure, volume, and osmolality through vagal afferents. These signals are integrated throughout the brain to regulate salt intake accordingly.

### 3.2. Endocrine Mediators of Sodium Appetite

In addition to being directly influenced by plasma osmolality and volume, the neural circuitry regulating sodium appetite also has intricate and reciprocal connections with endocrine systems that allow for the maintenance of sodium balance. Renin, the initiator of the RAAS, is released from kidney juxtaglomerular cells in response to increased sympathetic nerve activity (SNA) to the kidney, epinephrine release from the adrenal medulla, hypovolemia, or sodium depletion [86,87,88]. RAAS activation results in increased levels of circulating ANGII (a peptide hormone) and aldosterone (a mineralocorticoid produced within the adrenal gland), which serve as potent mediators of body fluid and blood pressure homeostasis [89]. In states of sodium depletion, circulating levels of ANGII and aldosterone are increased and their synergistic role in sodium appetite is established [90,91,92,93]. Studies conducted by Richter revealed that adrenalectomized rats, which do not produce adrenal hormones and as a result exhibit a sodium insufficiency due to decreased sodium retention, demonstrate a robust sodium appetite [94]. Richter’s experiments introduced a role for ANGII in sodium appetite, as adrenalectomy results in increased levels of circulating ANGII [95], however, the mechanism by which ANGII stimulates sodium appetite would not be understood for decades. Adrenalectomized rats treated with a low dose of deoxycorticosterone (DOC), a precursor for aldosterone, demonstrate an attenuation in sodium intake, most likely due to the restoration of sodium retention by aldosterone [96]. However, treatment with a higher dose of DOC in adrenalectomized and intact rats increases sodium intake [96], suggesting high levels of aldosterone stimulate sodium appetite. As such, ANGII was thought to stimulate sodium appetite indirectly through the release of aldosterone; however, its direct actions were made clear once administered into the brain. Central administration of ANGII increases water and salt intake [97]. Additionally, central administration of ANGII receptor antagonists attenuates water and/or salt intake induced by different challenges to fluid or electrolyte balance to stimulate thirst or sodium appetite [98,99,100]. These studies support a role for central ANGII in sodium appetite.

**Figure 2:**
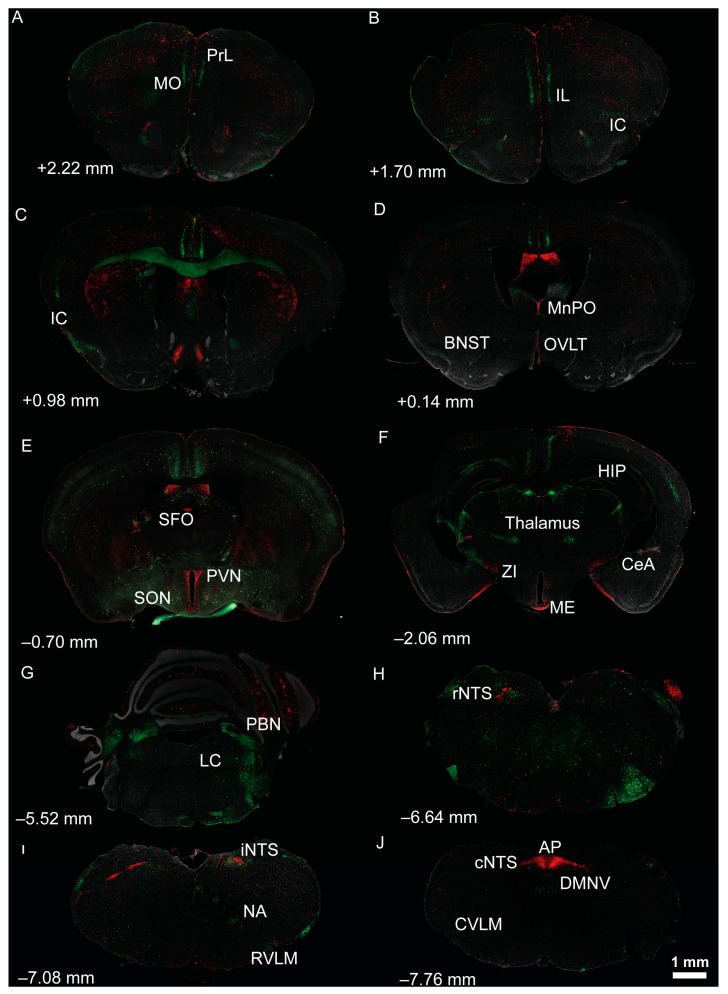
Localization of tdTomato and eGFP fluorescence throughout brain regions involved in salt intake and/or cardiovascular regulation of dual AT1aR-tdTomato/AT2R-eGFP reporter mice. (**A**–**J**) Low magnification coronal sections through selected (**A**–**F**) forebrain and (**G**–**J**) hindbrain regions of a male dual reporter mouse. AT1aR-tdTomato fluorescence is red; AT2R-eGFP fluorescence is green. Approximate distance from bregma, in accordance with the mouse brain atlas (Franklin and Paxinos, 2007), is noted in the lower left of each image. AP, area postrema; BNST, bed nucleus of the stria terminalis; CeA, central amygdala; cNTS, caudal nucleus of the solitary tract (NTS); CVLM, caudal ventrolateral medulla; DMNV, dorsal motor nucleus of the vagus; HIP, hippocampus; IC, insular cortex; IL, infralimbic prefrontal cortex; iNTS, intermediate NTS; LC, locus coeruleus; ME, median eminence; MnPO, median preoptic nucleus; MO, medial orbital prefrontal cortex; NA, nucleus ambiguous; OVLT, organum vasculosum of the lamina terminalis; PBN, parabrachial nucleus; PFC, prefrontal cortex; PIT, pituitary; PVN, paraventricular nucleus of the hypothalamus; PrL, prelimbic prefrontal cortex; rNTS, rostral NTS; RVLM, rostral ventrolateral medulla; SFO, subfornical organ; SON, supraoptic nucleus; ZI, zona incerta.

Thus, it has been long understood that ANGII and aldosterone act centrally to stimulate sodium appetite [54,100,101,102,103,104], but it was not until recently with the establishment of advanced techniques that we could start to elucidate their central mechanisms. ANGII exerts its influence by acting primarily at angiotensin type-1 (AT1R) and type-2 (AT2R) receptors, which are thought to elicit opposing effects [105,106]. Consistent with this, sodium homeostasis and blood-pressure regulation in mice deficient of AT1R exhibit decreased sodium retention and blood pressure [107] while mice deficient of AT2R exhibit increased sodium retention and blood pressure [108]. These receptors are localized to neurons in various brain regions involved in sodium and cardiovascular homeostasis [109,110,111,112] and Figure 2 highlights the expression of these receptors throughout brain areas that are involved in these processes. Although AT2R(s) are localized to numerous brain regions involved in sodium appetite, the role of this receptor sub-type in the neural control of sodium homeostasis is not yet established [100,113]. On the other hand, there is ample evidence that ANGII regulates sodium appetite by stimulating AT1R within various brain regions [112]. Circulating ANGII acts on AT1R expressed in circumventricular organs, primarily the OVLT and SFO. Fitts and Masson [114] demonstrated that ANGII administered directly into the OVLT or SFO of rats stimulates water intake, while OVLT administration also stimulates NaCl intake. To confirm a role for the OVLT in ANGII-induced NaCl intake, rats were chronically treated orally with a low-dose of captopril, an ACE inhibitor that does not readily cross the blood brain barrier, and stimulates water and NaCl intake by increasing central ANGII. Rats were then administered captopril into the OVLT or SFO, with administration into the OVLT decreasing NaCl intake relative to administration into the SFO. This suggests different roles for AT1R activation within the OVLT and SFO. Activation of SFO neurons by administration of clozapine *N*-oxide (CNO) targeting designer receptors exclusively activated by designer drugs (DREADDS) stimulated sodium appetite through activation of ANGII- and NaCl-responsive neurons [115]. This supports the idea that the SFO senses changes in [Na^+^] to regulate sodium appetite. In addition to hormonal actions, there is some evidence that ANGII acts as a neurotransmitter [116,117,118,119,120] and is therefore capable of stimulating AT1R in areas within the blood–brain barrier. The SFO has dense angiotensin-sensitive projections to the BNST [52,121] and PVN [117,118]. Optical excitation of these projections increases salt intake in dehydrated-mice, while optical inhibition decreases salt intake in sodium-deplete mice [52].

Another mechanism by which ANGII influences sodium homeostasis is by increasing circulating aldosterone levels. To stimulate sodium appetite, aldosterone binds to mineralocorticoid receptors located within the central amygdala, septum, hippocampus, and NTS [122,123,124]. A critical component of aldosterone-sensitivity to the mineralocorticoid receptor is expression of the enzyme 11-*β*-hydroxysteroid dehydrogenase type 2 (HSD2). HSD2 inactivates glucocorticoids to prevent their binding to the mineralocorticoid receptor, thus allowing aldosterone to bind. Within the last decade, a population of HSD2-expressing neurons in the NTS (NTS^HSD2^) have been at the forefront of aldosterone-induced sodium-appetite regulation. NTS^HSD2^ neurons are activated by sodium appetite and inactivated upon ingestion of NaCl [125]. Mice expressing excitatory or inhibitory DREADDS exclusively in NTS HSD2-expressing neurons were stimulated with CNO and exhibited changes in salt intake. Excitation of NTS^HSD2^ neurons drives sodium appetite, while inhibition mildly attenuates salt intake [126]. Surprisingly, conditional knockout of NTS^HSD2^ also produces sodium appetite, suggesting that they are important for sensing changes in blood volume and sodium status to respond accordingly [127]. Interestingly, these neurons respond to sodium depletion with pacemaker-like activity and can produce a rapid sodium appetite in conjunction with ANGII [93]. Collectively, these studies indicate NTS^HSD2^ neurons are necessary and sufficient for sodium appetite.

On the other end of the spectrum, is oxytocin, which is perhaps most well-known for its various roles in social behavior, reproduction, and childbirth [128]. As mentioned above, oxytocin is released into the blood stream by way of projections from PVN and SON to the posterior pituitary and a potent stimulus for this secretion is the sensation of hyperosmolality in the bloodstream. Oxytocin then plays an important role in restoring sodium homeostasis, in part, by acting at the kidney to promote natriuresis [128]. Of particular relevance here, many studies have also suggested a role for oxytocin in the satiation of sodium appetite and reduction in sodium intake [129,130,131,132,133]. In addition to secreting oxytocin into the bloodstream, PVN oxytocinergic neurons are also capable of releasing oxytocin centrally via projections within the brain—some of these central projections are important for oxytocin’s modulatory role in sodium appetite. ANGII may serve as another stimulus for oxytocin release from the PVN, as AT1R are robustly expressed within the PVN. In support of this, ANGII administered into the ventricle neighboring the PVN increases plasma oxytocin in rats [134]. Additionally, the PVN receives input from angiotensinergic SFO neurons [118]. Therefore, ANGII may bind AT1R on PVN-neurons to stimulate the release of oxytocin to inhibit sodium appetite. These actions of ANGII in the PVN could serve as a mechanism to regulate the stimulatory effects of ANGII on salt intake.

Oxytocin’s physiological effects are exerted via the oxytocin receptor (Oxtr) which is expressed in various peripheral organ systems and the brain [135]. What initially sparked the idea that oxytocin is involved in sodium-appetite regulation were observations that plasma oxytocin levels were low in sodium-deficient rats and their sodium appetite was inhibited by treatments that stimulated oxytocin release [129,136]. This inverse relationship between oxytocin levels and salt intake was observed in various models of sodium appetite [129,136]. Mice genetically modified to lack the oxytocin receptor demonstrated increased salt intake under water deprived conditions [137], but not ad libitum water access conditions [138]. This implies that oxytocin is involved in regulating salt intake during states of compensation for changes in volume and sodium balance. The actions of oxytocin in sodium appetite were determined to be mediated by the brain. This is supported by studies in which neither systemic administration of oxytocin nor blockade of peripheral Oxtr reduced salt intake in rats rendered hypovolemic by way of subcutaneous injection of polyethylene-glycol (PEG) [129]. Additionally, administration of an Oxtr antagonist into the cerebral ventricles prevented inhibition of salt intake in PEG-induced hypovolemic rats treated with systemic naloxone, an opioid antagonist, to stimulate oxytocin release [139]. A population of Oxtr-expressing neurons within the PBN that receive oxytocinergic projections from the PVN has recently been implicated in sodium appetite. Inhibition of these neurons through DREADDs increased salt intake in dehydrated, hyperosmotic, and ad libitum water access conditions, while activation did not alter salt intake following sodium depletion [133]. This suggests different populations of Oxtr-expressing neurons throughout the brain may play distinct roles in salt appetite and fluid homeostasis.

Atrial natriuretic peptide (ANP) has also been shown to play an inhibitory role in sodium appetite. ANP is primarily produced and released by the atria of the heart in response to elevated blood volume or pressure [140]. Binding sites for ANP have been identified within brain regions involved in cardiovascular and fluid homeostasis regulation [141]. ANP administered into the third ventricle of sodium-deplete rats attenuated salt intake [142,143]. Like oxytocin, increased ANP plasma levels are associated with sodium appetite satiety [144].

Stress hormones, particularly glucocorticoids and catecholamines, have also been implicated in regulating sodium appetite. Activation of the hypothalamic–pituitary–adrenal (HPA) axis, described in Section 4.2, results in increased levels of circulating glucocorticoids, which, in turn, potentiate the effects of aldosterone on sodium appetite [96,145,146,147]. On the other hand, norepinephrine and phenylephrine, which has similar actions to epinephrine, appear to play more of an inhibitory role in sodium appetite. Central administration of norepinephrine or phenylephrine decreases salt intake in rats stimulated by DOC, chronic intracerebroventricular renin, or sodium depletion [148,149,150]. Results from these studies suggest that there are region and adrenergic receptor type specific effects of norepinephrine and phenylephrine.

The mechanisms underlying sodium-appetite regulation are complex and becoming more understood with advancing techniques. The behavior in sodium appetite is simple—consume salt to restore a sodium-deficit—and has been observed in various species [151,152]. Salt intake is tightly regulated to maintain sodium balance to meet physiological needs. However, it is not so simple in humans as we rarely encounter a sodium-deficit and consume sodium in excess of physiological needs.

### 3.3. Gustatory Mediation of Sodium Appetite

Sodium depletion shifts the appetitive-aversive balance of salt taste so that concentrations of NaCl that are normally aversive are now considered appetitive. These alterations in salt taste are critical for driving increased sodium intake observed in sodium appetite [2,153]. The mechanisms of normal salt-taste transduction are not well understood and, therefore, mechanisms underlying this shift with sodium depletion are unclear as well. Evidence supports changes in gustatory sensory coding and the hedonic value of sodium contribute to this shift in salt taste during sodium appetite and may occur at the level of the taste bud through central gustatory areas [2,78,154,155].

**Figure 3:**
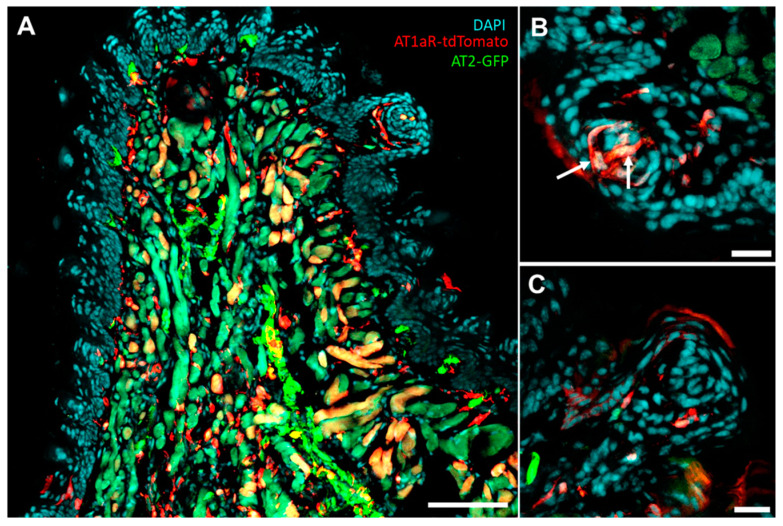
Representative images of coronal section of tongue from AT1aR-tdTomato/AT2R-GFP dual reporter mouse. (**A**) 20× image showing tdTomato and GFP are expressed throughout tongue tissue (scale bar = 100 uM). (**B**) 40× image depicting tdTomato expression in fungiform taste bud cells (scale bars = 20 uM). (**C**) 40× image depicting tdTomato and GFP expression near fungiform taste bud (scale bars = 20 uM).

Early on, it was proposed that taste receptor cells become more sensitive to NaCl with sodium depletion (due to adrenalectomy), making it easier for them to discriminate different concentrations of NaCl [156]. Taste receptor cells can receive hormonal regulation to modulate taste as they express receptors for various hormones. Of relevance, others have revealed that a subset of taste receptor cells, including type I cells, express the AT1R [157] and Oxtr [158]. Consistent with this, we have also observed AT1R expression within taste receptor cells of AT1aR-tdTomato/AT2R-GFP dual reporter mice (Figure 3). Collectively, these findings indicate ANGII and oxytocin, in addition to impacting salt appetite as described above, may also regulate sodium intake by acting on the taste bud to influence salt taste.

Electrophysiological recordings of gustatory nerves in rodents have provided in-sights into processes involving neural mediation of salty taste in sodium appetite. Whole nerve and single nerve fiber recordings from the chorda tympani conducted in rats maintained on a sodium-deficient diet indicate responsiveness to NaCl decreases [159,160]. The single nerve fiber recordings [159,160] determined that it was neurons that responded best to NaCl that were attenuated. Within the NTS, NaCl-best neurons are also less responsive to hypertonic NaCl, in agreement with [160], while sucrose-sensitive neuron responsiveness increases [154,161]. Rapid induction of need-free sodium appetite in rats pre-treated with DOCA and administered intracerebroventricular renin exhibit similar responses in the NTS [162]. However, sodium appetite stimulated by low-dose furosemide increased responsiveness to salt in NaCl-best neurons in the NTS of rats [163]. The inconsistency in responsivity of NaCl-neurons within the NTS suggest different models of sodium appetite (e.g., sodium-deficient diet versus furosemide), with different underlying mechanisms to stimulate salt intake, may alter taste activity differently.

The shift in NaCl-sensitive and sucrose-sensitive neuron activity suggests increased acceptance of typically aversive concentrations of NaCl and may underlie the change in its hedonic value that is observed. However, this is not supported by behavioral studies or studies using other methods to arouse sodium appetite. Furosemide-treated sodium-deplete rats with a conditioned taste aversion to sucrose do not exhibit an aversion to NaCl, while sodium-deplete rats with a conditioned taste aversion to NaCl do not express a sodium appetite [155]. This implies NaCl still tastes salty as the increased drive and preference for NaCl in the sodium-deplete state did not circumvent the conditioned taste aversion to NaCl. These inconsistencies demonstrate the mechanisms underlying the shift in salt taste hedonic value observed with sodium appetite remain unclear. While beyond the scope of this review, the shift in hedonic value toward consumption of typically aversive concentrations of NaCl during sodium-appetite may be mediated by mesocorticolimibic circuitry, including the nucleus accumbens, ventral pallidum, ventral tegmentum, and OFC [164].

Although the mechanisms mediating changes in salt taste during sodium depletion are not well understood, the role for salt taste in regulating sodium appetite is clear. Intake of hypertonic NaCl observed in sodium-deplete animals does not increase until changes in salt taste occur. In driving NaCl intake toward restoration of sodium balance, salt taste contributes to the satiation of sodium appetite. Investigations of taste and post-ingestional factors in the satiety of sodium appetite indicate oral ingestion of NaCl more rapidly induces satiation compared to stomach loading of NaCl [165,166]. The satiation of sodium appetite by oral ingestion of NaCl is mediated by neural circuitry involved in regulating sodium intake. The pre-locus coeruleus (pre-LC), located within the hindbrain, receives projections from the NTS relaying information pertaining to the state of the internal milieu (interoception). Pre-LC neurons involved in regulating sodium appetite are inhibited by oral ingestion of NaCl and not gastric loading with NaCl, suggesting they are involved in taste mediated satiety of sodium appetite [167]. The relationship between salt taste and sodium appetite supports the notion that taste can be mediated by physiological state.

## 4. Neural and Hormonal Control of Blood-Pressure

The mechanisms and organ systems that are involved in salt intake contain several points of overlap with the processes that are involved in blood-pressure regulation. Thus, it is perhaps not surprising that mechanisms that are traditionally considered for their roles in maintaining blood volume and pressure, also impact sodium appetite and possibly taste. Blood pressure, which is dictated by cardiac output, peripheral vascular resistance and blood volume, is regulated by mechanisms that involve neural, endocrine, cardiovascular and renal systems and are intricately-related to those that mediate sodium intake and are discussed above [168,169,170,171,172,173]. What was not discussed in detail above and is also of particular importance for the regulation of blood pressure, is the autonomic nervous system. The autonomic nervous system is comprised of parasympathetic and sympathetic arms that, generally, act in opposition to one another to maintain physiological functions. The parasympathetic nervous system primarily modulates heart rate and is most active at rest, while the sympathetic nervous system controls both cardiac output and peripheral vascular resistance and is activated during states of real or perceived stress. While both parasympathetic and sympathetic limbs of the autonomic nervous system are active at any given time, the balance between the opposing limbs shifts based on the internal and external conditions and this balance is vital for determining blood pressure. The endocrine factors discussed above then also mediate blood pressure homeostasis by way of acting peripherally to influence vasoconstriction and fluid volume and centrally to impact behavior and autonomic outflow.

### 4.1. Neural Circuitry Underlying Blood-Pressure Control

The neural circuitry that is involved in cardiovascular homeostasis has been reviewed in detail elsewhere [171,174,175], and involves parallels with the circuitries that mediate sodium appetite and taste (Figure 2). Similar to the regulation of sodium appetite, the neural circuitries involved in controlling blood pressure depends on the ability to detect changes in the internal milieu. This is accomplished, in part, by sensory CVOs also involved in sodium appetite, including the SFO, OVLT, and AP, that are highly interconnected with other brain regions involved in cardiovascular regulation, such as the NTS, PBN, LC, PVN, SON, and BNST [49,50,51,53,176]. Plasma osmolality not only impacts salt appetite but also blood-pressure regulation. Blood pressure is regulated, in part, by the detection of changes in [Na^+^] within the brain by osmosensitive neurons located near the anteroventral third ventricle region (AV3V) [177,178,179], including the SFO, OVLT, and MnPO. As discussed with sodium appetite, osmosensitive neurons express various [Na^+^]-sensitive channels, including TPRV1, TRPV4, and Na_x_. Projections from the AV3V modulate blood pressure by influencing autonomic and neuroendocrine activity [180,181,182]. The PVN, a major integrative site mediating the interpretation of and responses to internal and external stressors, receives inputs from AV3V neurons and plays a critical role in blood-pressure regulation through control of neuroendocrine release and sympathetic activity [180,183,184,185,186,187,188]. As with sodium appetite, neuropeptides (e.g., oxytocin and vasopressin) produced by the PVN and SON in response to changes in plasma osmolality or blood volume can impact the neural circuitry that mediates blood pressure.

Much like its role in impacting sodium intake and appetite, the NTS also plays a critical role in integrating information from the periphery and relaying this information within the brain to regulate blood pressure. Information from baroreceptors located within the carotid sinus and aortic arch send signals to the NTS through the glossopharyngeal nerve and vagus nerve, respectively [69,70]. Baroreceptors are mechanosensitive neurons [189] that sense changes in vascular stretch and relay information regarding blood pressure and volume. Inputs from arterial baroreceptive afferents are integrated by the NTS and then relayed to regions involved in regulating blood pressure and volume. NTS hindbrain projections that are particularly important for the control of the arterial baroreceptor reflex (baroreflex), a rapid negative feedback loop that modulates autonomic control of blood pressure, are those to the nucleus ambiguous (NA) and caudal ventrolateral medulla (CVLM) [190,191,192]. The NA contains parasympathetic cardiac vagal motor neurons important in regulating heart rate [193]. The CVLM influences sympathetic tone indirectly through inhibitory projections to the rostral ventrolateral medulla (RVLM) [191]. Unlike the CVLM, the RVLM has direct projections to sympathetic preganglionic neurons to control SNA [194,195,196]. Increased blood pressure induces a reflexive decrease in heart rate and sympathetic outflow to lower blood pressure, while a reduction in blood pressure induces opposite effects. Ventral to the NTS is the dorsal motor nucleus of the vagus (DMNV), which receives inhibitory connections from the NTS [197,198,199,200] and contains parasympathetic cardiac vagal motor neurons important in regulating contractility of the heart [201,202,203]. Moreover, direct baroreceptor afferent input has been observed in the DMNV and this area receives information from various brain regions involved in blood-pressure regulation, including the NTS and PVN [204,205,206].

Projections from the NTS to the PBN are also important for integrating signals pertaining to blood pressure and volume, fluid, and electrolyte homeostasis. The PBN integrates these inputs and transmits information throughout the brain to mediate neuroendocrine and autonomic activity to restore homeostasis [51,77,207,208,209,210]. The LC, adjacent to the PBN, has also been implicated in blood-pressure control. Rats subjected to hemorrhage to induce hypovolemia exhibited increased c-Fos expression in the posterior LC, while rats subjected to extracellular volume expansion did not [211,212]. These results suggest the LC may be involved in controlling mechanisms to compensate for volume loss. However, the LC can also influence blood pressure through mechanisms that involve the sympathetic nervous system [213].

One area receiving input from the PBN, and other areas, is the insular cortex which serves as an integrative hub involved in interoception, sensory processing (e.g., gustatory and cardiovascular), and autonomic control [214]. The IC has been implicated in blood-pressure control [215] and regulating cardiovascular responses to acute stress [216,217]. The PVN also receives information pertaining to blood pressure and volume through projections from the NTS and PBN [207,218,219,220]. Baroreceptor activity is important for PVN neuroendocrine release (e.g., vasopressin) and sympathetic responses to changes in blood pressure. Like the RVLM, the PVN contains direct projections to sympathetic pre-ganglionic neurons [221]. The PVN also modulates SNA through projections to the RVLM [222]. PVN projections to sympathetic pre-ganglionic neurons and the RVLM are inhibited by increases in blood pressure, further supporting the notion that these projections receive information from baroreceptors [223]. The PVN is, in turn, also capable of modulating the baroreflex through projections to the NTS [224]. Electrical stimulation of PVN neurons attenuated the firing rate of NTS pressor neurons and inhibited the baroreflex.

Additionally, corticolimbic areas that are sensitive to stress and contribute to behavioral responses to stress, including the mPFC, amygdala, and hippocampus, descend upon regions involved in cardiovascular regulation to modulate blood pressure. Regions of the BNST and hypothalamus, as well as the NTS and VLM, receive input from the mPFC, amygdala, and hippocampus to modulate autonomic control [225,226]. Blood pressure and heart rate increase in response to acute perceived stressors [227,228,229,230,231]. The baroreflex is inhibited by corticolimbic descending projections to the NTS to prevent a reflexive decrease in heart rate following a stressor-induced increase in blood pressure [232,233]. The PVN also receives input from corticolimbic regions important for modulating blood pressure in response to perceived stress. These circuitries are well documented in the literature [225,234,235,236,237,238,239].

As with sodium appetite, the brain senses and receives information pertaining to changes in the internal milieu to regulate blood pressure. Changes in plasma osmolality, pressure, and volume are detected by osmoreceptors or baroreceptors. This information is integrated by various brain regions and relayed accordingly to regulate autonomic and behavioral responses. As will be discussed below, changes in blood pressure can alter salt intake. Which is not surprising as the neural circuitry involved in regulating cardiovascular homeostasis involves numerous points of overlap with the circuitries that mediate sodium appetite and taste.

### 4.2. Endocrine Mediators of Blood-Pressure Control

The neural circuitry involved in blood-pressure regulation is comprised of pathways to initiate compensatory mechanisms to maintain survival (e.g., baroreflex or “fight-or-flight” response). As such, the neural circuitry regulating blood pressure also has intricate and reciprocal connections with endocrine systems that similarly allow for the maintenance of cardiovascular homeostasis in various situations. As previously described, renin release is stimulated by increased SNA to the kidney, epinephrine release from the adrenal medulla, hypovolemia, or sodium depletion [86,87,88]. Renin release results in increased levels of circulating ANGII and aldosterone that then act in the brain and periphery to increase blood pressure, as well as stimulate sodium appetite. Aldosterone’s influence on blood pressure is most understood from a renal perspective, in that it stimulates water and sodium reabsorption by the kidney to increase blood volume and pressure. Aldosterone can impact blood pressure centrally by acting on mineralocorticoid receptors within the brain to stimulate salt intake, as seen in animal models utilizing DOC and DOCA, and alter sympathetic activity [240,241,242,243]. As with the stimulation of sodium appetite, aldosterone, and ANGII act together centrally to increase blood pressure. When injected directly into the brain, ANGII increases blood pressure [244]. In the brain, ANGII exerts pressor effects by acting on AT1R found in various regions involved in cardiovascular regulation [109,110,111,245]. ANGII can modulate the baroreflex by acting on AT1Rs in the AP and NTS [246,247,248]. ANGII acts on AT1Rs in other regions to modulate neural signaling, neuroendocrine release, and SNA to alter blood pressure. Circulating ANGII can act on AT1Rs expressed in CVOs, including the OVLT and SFO, resulting in increased blood pressure [187,249] and stimulation of sodium appetite. ANGII stimulation of AT1R in the RVLM [250,251] and MnPO [252] increases activity in neurons involved in blood-pressure control, as well as salt-sensing neurons in the MnPO. The PVN has robust AT1R expression and receives angiotensin-sensitive projections from various regions to mediate sympathetic and neuroendocrine activity in response to ANGII [187,253,254,255]. For example, AT1R are expressed on PVN corticotropin releasing hormone (CRH) neurons that serve as a relay to and activate pre-autonomic neurons via CRH release [188].

As mentioned, baroreceptor activity is important for neuroendocrine release in response to changes in blood pressure. Decreases in blood pressure induce vasopressin release from PVN and SON neurons that is largely dependent on signals from baroreceptors [256]. The central and peripheral actions of vasopressin in blood-pressure regulation are well documented [172,257]. Oxytocin has various actions, including inhibition of sodium appetite, and has been implicated in blood-pressure regulation and these actions are reviewed in detail in [258]. Another key mechanism of the PVN in regulating cardiovascular homeostasis to stressors is the HPA axis. HPA-axis activity is strongly related to activity in limbic areas involved in emotional and behavioral responses including the hippocampus, prefrontal cortex, and amygdala [234,259]. Activation of the HPA axis stimulates the release of CRH from PVN neurons [260]. CRH acts on the anterior pituitary to stimulate the release of adrenocorticotropic hormone (ACTH) into the systemic circulation. ACTH stimulates the adrenal cortex to synthesize and release glucocorticoids, cortisol in humans and corticosterone in rodents. Similar to their potentiating effects of aldosterone in sodium appetite, glucocorticoids potentiate the effects of catecholamines and vasoconstrictors on the vasculature to increase blood pressure. Rats treated with oral dexamethasone displayed potentiated pressor responses to norepinephrine [261]. DOC-treated rats exhibited greater blood pressure responses to intravenous ANGII [262]. Studies performed in humans are consistent with those in rats [263,264]. Additionally, HPA activation increases the release of epinephrine from the adrenal medulla [265], which influences blood pressure through a variety of mechanisms that involve activation of adrenergic receptors throughout the body [266,267,268]. ANP is another hormone that is released that is dependent on changes in blood volume and pressure and plays a role in sodium appetite and blood-pressure control.

Disruptions to homeostasis, real or perceived, stimulate the release of endocrine and neuroendocrine hormones important to controlling blood pressure. Endocrine mechanisms contribute to the maintenance of cardiovascular homeostasis by regulating physiological (e.g., heart rate) and behavioral (e.g., salt intake) responses in various situations. Changes in blood pressure, volume, and/or osmolality control the release of ANGII, aldosterone, vasopressin, and oxytocin while stress-induced stimulation of the HPA-axis leads to the production and release of glucocorticoids. Each of these hormones may act centrally or peripherally to exert their effects and ultimately restore blood pressure.

## 5. Mechanisms Underlying Integration of Cardiovascular Homeostasis and Salt Intake

As alluded to throughout this review, the neural circuitry that is involved in salt intake contains several points of overlap with the circuitry involved in blood-pressure regulation. Thus, it is perhaps not surprising that mechanism(s) that are traditionally considered for their role(s) in maintaining blood volume and pressure, also impact sodium appetite and possibly taste. One such mechanism is baroreceptors.

Baroreceptor mediation of sodium appetite is supported by studies in which baroreceptors are surgically manipulated and salt intake is assessed. Atrial balloon studies involve the surgical implantation of a balloon into the superior vena cava-right atrial junction. Inflation of the balloon mechanically stimulates baroreceptors and simulates increased blood pressure or volume to the brain. Sodium-deplete and DOC-treated rats demonstrated decreased salt intake upon balloon inflation, with a rebound in intake following deflation of the balloon [269]. Atrial balloon inflation shows a similar effect in rats treated with furosemide and captopril to induce sodium appetite [270]. In this study, they also determined that balloon inflation following induction of an acute sodium appetite altered c-Fos expression in brain regions associated with sodium-appetite regulation. Specifically, c-Fos was increased in the PBN and CVLM and decreased in the OVLT and SFO [270].

Sinoaortic denervation (SAD) studies, in which baroreceptor afferent input to the brain is removed, also provide insight into baroreceptor regulation of sodium appetite. SAD rats made sodium-deplete by furosemide exhibit decreased salt intake [271], and under normal physiological conditions, SAD rats also consumed significantly less salt than controls [272]. Collectively, these studies support the idea that neural input from baroreceptors are important for salt intake regulation and normal expression of sodium appetite.

There are various potential sites throughout the brain that may contribute to changes in salt intake observed with baroreceptor manipulation. Salt taste and baroreceptor sensory afferents enter the brain in the rostral and caudal NTS, respectively. Projections from the caudal NTS to the rostral NTS may alter gustatory information at the first input from the periphery [76]. Another early relay point for gustatory and cardiovascular sensory information is the PBN, which also plays an important role in salt intake regulation. The PBN may serve as a site of integration of gustatory and cardiovascular information to regulate salt intake. Endocrine and neuroendocrine factors released in response to baroreceptor stimulation act in some of the same brain regions to regulate blood pressure or salt intake, such as the SFO and PVN. The exact underlying mechanism(s) behind cardiovascular status influencing salt intake remain unclear, and more work needs to be completed to understand how blood pressure status impacts salt intake.

## 6. Stress-Induced Dysregulation of Salt Intake

Thus far, we have described mechanisms that regulate sodium intake under normal physiological conditions. Many of these mechanisms are altered upon exposure to stress, external or internal. Stress is defined as a real or perceived threat to homeostasis and can occur acutely, repeatedly, or chronically [273,274,275,276]. While stress is not a disease, per se, the psychological and physiological effects of stress manifest to other diseases, including cardiometabolic diseases (CMDs), such as obesity and hypertension [277,278,279]. Additionally, environmental, and physio-logical challenges (e.g., overconsumption of salt, disease pathophysiology, etc.) can be perceived as stressful as they threaten homeostasis. Stress-related diseases, including CMDs, are highly prevalent and on the rise worldwide [280,281,282]. Experimental evidence implies that psychological stress, hypertension, and obesity are associated with increased salt intake [283,284,285,286]. We will discuss salt intake in each condition and highlight some of the potential mechanism(s) that become impaired to contribute to increased intake.

### 6.1. Psychological Stress

You have probably found yourself reaching for a salty snack when feeling stressed and there could be a reason for that. Many studies have investigated the effect of psychological stress on salt intake in humans and small mammals, with most reporting increased intake. An acute model of psychological stress in rodents is restraint, in which animals are placed in a restrainer for a pre-defined duration of time. Relative to their baseline intakes, hamsters exhibit increases in salt intake after restraint-stress [287]. Other studies have utilized chronic models in rodents and rabbits to understand the effects of chronic stress on salt intake. A chronic model of social stress in rodents involving the introduction of and long-term cohabiting with an “intruder”. Ely et al. [288] applied this model to rats and measured daily salt intakes. Male rats exhibited a significant stress-induced increase in daily salt intake. Furthermore, exposure to stress early in life can also have long-lasting effects that impact salt intake. Rats exposed to sodium depletion or maternal separation as pups demonstrated increased salt intake in adulthood [289]. The implication is that early life stressors may contribute to shaping sodium preference in adulthood.

Psychological stress is known to increase circulating glucocorticoids [290,291,292,293]. Chronic administration (1 week) of ACTH systemically or CRH intracerebroventricularly simulates this aspect of chronic stress and also increases daily salt intake in rabbits [294]. When considering the interaction between glucocorticoids and salt intake it is worthwhile noting that these steroid hormones are important ligands for the mineralocorticoid receptor, which is expressed in and mediates sodium appetite by way of the NTS. Therefore, it is reasonable to hypothesize that the NTS neurons that express the mineralocorticoid receptor may be involved in altered sodium intake during psychological stress; however, it is also important to reiterate that the presence of HSD2 on these neurons biases the receptor away from glucocorticoids to favor aldosterone binding (see Section 3.2). With this in mind, another relevant consideration is that psychological stress is associated within autonomic imbalance and increased activity of the RAAS that may mediate the increased sodium intake [91,93,295,296].

Evidence from human studies investigating stress and salt intake has been less consistent. This may be due to methodology used and some of the caveats associated with human research. Numerous studies utilizing acute stressors (performing a math task with harassment, preparing a speech with expectation of performing, or attempting to solve unsolvable anagrams) in humans show no change in self-reported salt intake [297,298,299]. However, exposure to acute stress reduced salt-taste sensitivity and thresholds [300,301]. Stressors used in the latter studies included public speaking, mental arithmetic, and a cold pressor test or completion of a color-word interference test followed by a cold pressor test. Testing of gustatory sensitivity and thresholds may provide more insight on acute stress-induced changes in salt intake that self-report measures of salty food intakes. Few studies have looked at the effect of chronic psychological stress on salt intake in humans. One study investigated food choices in young adults in relation to reported feelings of stress. Reported consumption of highly salty snack foods increased with stress [283].

Studies in mice and rats suggest salt intake may have a ‘stress-dampening’ effect, such that acute increases in plasma sodium concentration due to increased salt intake induces compensatory mechanisms that decreases reactivity to psychological stressors. In support of this, animals administered hypertonic NaCl (2.0 M) prior to exposure to restraint-stress exhibit decreased HPA activation as reflected by an attenuated surge in corticosterone compared to animals administered isotonic NaCl. This implies acute increases in plasma sodium concentration reduces responsivity to the stressor (restraint) [302,303]. Further, acute administration of hypertonic NaCl increased neural activation in PVN oxytocinergic neurons and decreased neural activation in PVN CRH neurons [304,305]. This suggests the ‘stress-dampening’ effect of salt intake may be mediated by stimulation of oxytocin and inhibition of CRH neurons in the PVN and may contribute to increased salt intake observed with stress.

Psychological stress is a major risk factor for the development of hypertension and obesity [306,307,308,309,310,311]. We propose that this risk may be mediated by increased salt intake in response to stress. As we have already highlighted, stress hormones can induce salt intake [146,147,294]. HPA-axis hyperactivity and excess glucocorticoids, and other stress hormones, are also reported in obesity and hypertension [312,313,314,315,316,317,318]. Thus, it is possible that dysregulation of stress mechanisms may contribute to increased salt intake observed in obesity and hypertension.

### 6.2. Obesity

The worldwide prevalence of obesity, defined as abnormal or excessive fat accumulation that may impair health, has nearly tripled in the last 50 years [281]. In addition to psychological stress, other factors contribute to rising obesity levels, such as sedentary lifestyle and diet. Like sodium appetite, obesity seems to alter salt palatability resulting in increased consumption of salty foods. As such, the relationship between body mass index (BMI) and taste perception has been investigated in humans and animals. Park et al. [286] assessed taste in obese and lean adults using two different methods, electrogustometry and chemical taste tests. Electrogustometry depends on delivery of electrical currents to areas of the tongue to stimulate taste buds and recognition of the stimulation. Obese individuals exhibited higher recognition thresholds to electrical stimulation on the anterior and posterior tongue, while only those to the posterior tongue were significantly higher. Chemical taste tests were performed by applying solutions representing the basic taste qualities at various concentrations to the tongue and the minimum concentration at which a taste was detected was defined as the threshold. Obese individuals had higher thresholds across all taste qualities, while only the threshold for salt taste was significantly higher. In another study, taste was assessed using the “taste strips” test in which a paper saturated with solutions representing the basic taste qualities at different concentrations is applied to the tongue. The lowest concentration at which taste is detected served as the threshold. Consistent with the previous study, they observed higher taste thresholds for salt in obese individuals [319].

While these studies support an association with obesity and salt taste sensitivity, humans often consume salt at suprathreshold concentrations. To understand how obesity impacts sodium consumption in humans, we must look at changes in taste to suprathreshold concentrations of salt. Suprathreshold assessment of salt taste involves presenting a participant with solutions containing varying high concentrations of salt and asking them to identify which they consider to be intolerably salty. Studies investigating suprathreshold perception in obesity have been inconsistent. Li et al. [11] performed salt suprathreshold tests and functional brain imaging in lean and obese individuals to determine changes with obesity. Participants were asked to sip the taste solution and hold it in their mouth for 5 s. Suprathresholds for salt were increased in obese individuals suggesting that they find higher concentrations of salt more palatable than lean individuals. PET/CT scans revealed obese individuals had higher activity in the insular cortex, orbitofrontal cortex, and parrahippocampus following buccal administration of the taste solution (200 mmol/L NaCl). As previously described, the gustatory cortex is located within the insular cortex. One caveat of this study is that participants with hypertension or elevated blood pressure were included, making it difficult to determine if these effects are due to obesity or hypertension. While these studies suggest decreased sensitivity for salt taste based on suprathreshold assessment, others have observed increased sensitivity. Hardikar et al. [320] performed suprathreshold assessments in lean and obese individuals in which taste solutions were administered to the anterior part of the tongue. Obese individuals tended to rate the same concentration of a taste solution as significantly more intense than lean individuals. The inconsistency between studies in investigating suprathreshold perception may be due to a difference in methodology.

One contributing factor to altered salt taste observed in obesity may be the number of taste buds. Mice with diet-induced obesity were found to have significantly fewer taste buds than mice on a normal diet [321]. Changes in taste responses have also been reported within the brain. As previously discussed, various brain regions exhibited higher activity following buccal administration of a salt solution [11]. However, this study does not provide insight into the simultaneous neural changes in the brain with salt taste stimulation. EEG recordings were performed in lean and obese individuals and gustatory-event related potentials were assessed in response to oral stimulation of a high or low concentration salt solution, delivered through a computer-operated gustometer. Obese and lean individuals demonstrated a similar processing of taste within the gustatory network, however activity diminished sooner in obese individuals [322]. This shorter neural representation of taste information could be linked to weaker taste sensitivity. Single-cell electrophysiological recordings performed in the NTS of awake, behaving diet-induced obese and lean rats are consistent with this. Obese rats demonstrated longer latencies, smaller magnitudes, and shorter durations of taste evoked responses in NTS cells [323]. These changes in salt taste responses are consistent with those observed in sodium-deplete rodents that demonstrate decreased responsiveness to NaCl from the chorda tympani [159,160] and within the NTS [154,161] and PBN [78].

Endocrine and neuroendocrine factors may also contribute to dysregulation of salt intake in obesity. RAAS activity is elevated in obesity and contributes to elevated levels of ANGII and aldosterone [324,325]. ANGII and aldosterone stimulate salt intake. Oxytocin, which plays an inhibitory role in salt intake, is also altered in obesity [326]. Oxytocin release and systemic oxytocin have been shown to be decreased in obesity [327,328].

With the prevalence of obesity on the rise, much work is dedicated to understanding its physiological consequences. Taste changes in obesity have been reported by many, while the evidence remains inconsistent regarding salt taste. Salt taste changes with obesity are particularly of interest due the relationship between salt intake and hypertension. Obesity and hypertension often go hand in hand [329,330], however, many studies investigating salt intake in obesity do not consider hypertension.

### 6.3. Hypertension

Chronic exposure to psychological stress and obesity are both often associated with co-morbid hypertension, which is a major risk factor for cardiovascular disease, the leading cause of death worldwide [280]. Due to the link between salt intake and hypertension, the relationship between salt taste and hypertension has long been of interest. Early comparisons of taste thresholds in normotensive and hypertensive individuals revealed hypertensive individuals had a significant reduction in their ability to taste salt [331,332,333]. This led the authors to deduce hypertensive individuals consume more salt because they cannot taste it as well. In support of this, a correlation between blood pressure and salt taste detection thresholds has been observed. Salt-taste acuity and discrimination tests were performed in normotensives. In salt-taste acuity tests, participants taste a series of solution pairs and determine which is saltier, while in salt-taste discrimination tests participants are presented with varying concentrations of salt solutions and asked to sort them based on intensity. A significant association between systolic blood pressure and sensitivity was observed, in that individuals with higher blood pressure had lower detection thresholds [334].

It is important to note that there are different forms of hypertension with different underlying causes and the studies discussed here focus on essential hypertension. Essential (primary) hypertension occurs independent of another medical condition, such as renovascular disease. In patients with essential hypertension, plasma renin activity may vary. Salt taste assessments measuring intensity and hedonic value performed in low-renin hypertensives, normal-renin hypertensives, and normotensives demonstrate such differences. Only low-renin hypertensives preferred salt solutions at various concentrations compared to normotensives [335]. In contrast, spontaneously hypertensive rats (SHRs), an animal model for essential hypertension with high-renin [336], also display alterations in salt-taste responses. Pereira et al. [337] investigated hedonic responses in SHRs and control rats by measuring orofacial responses to intraoral infusions of hypertonic saline under euhydrated conditions. SHRs exhibited more hedonic responses to intraoral infusion of saline and these responses were attenuated by intracranial administration of losartan, an ANGII receptor blocker. This suggests salt-taste changes observed in this model of hypertension are attributed to increased ANGII signaling in the brain.

The prevalence of hypertension [338], and CMDs [339], increases with age. Taste perception is susceptible to decline with age [340,341] and changes in salt taste with age have been observed in humans and rodents [342,343]. One study compared salt preference in young (age = 30–50 years) and older (age = 60–80 years) normotensive and hypertensive individuals in order to discriminate the influence of age and hypertension on salt preference and intake. Hypertensive individuals, regardless of age, preferred and consumed more salt than normotensives [285], suggesting hypertension more strongly influences salt taste and preference than aging.

Mechanism(s) involved in regulating blood pressure and salt intake are impaired with hypertension and may contribute to increased salt intake. Baroreflex impairment is observed in hypertension, as well as obesity, and could be the result of altered afferent signaling [344,345,346,347,348]. Impaired baroreceptor afferent input to the NTS may contribute to increased salt intake. Endocrine and neuroendocrine signaling is also dysregulated with hypertension. Increased RAAS activity in hypertension is well documented [349,350,351]. AT1R expression has been shown to be elevated in regions related to salt intake regulation in rodents including the NTS, PVN, and SFO [352,353]. Additionally, increased numbers of HSD2 neurons in the NTS has been observed in hypertensive rats [354]. Increased levels of ANGII and aldosterone and more sites for their action in hypertension may contribute to increased salt intake. Alternatively, oxytocin release, systemic oxytocin levels, and Oxtr expression in the PVN and NTS have been reported to be decreased in hypertension [355,356,357,358]. With less oxytocin and fewer sites of action in hypertension, there is less potential for inhibition of salt intake.

Understanding how hypertension increases salt intake is of obvious importance as salt intake can exacerbate the condition and increase the risk for cardiovascular disease. Many studies, in humans and animals, have reported elevated salt intake and changes in salt taste in hypertension. Discerning these mechanism(s) may provide a potential therapeutic target or increase the success of dietary salt reduction in treating hypertension.

## 7. Conclusions and Perspectives

**Figure 4:**
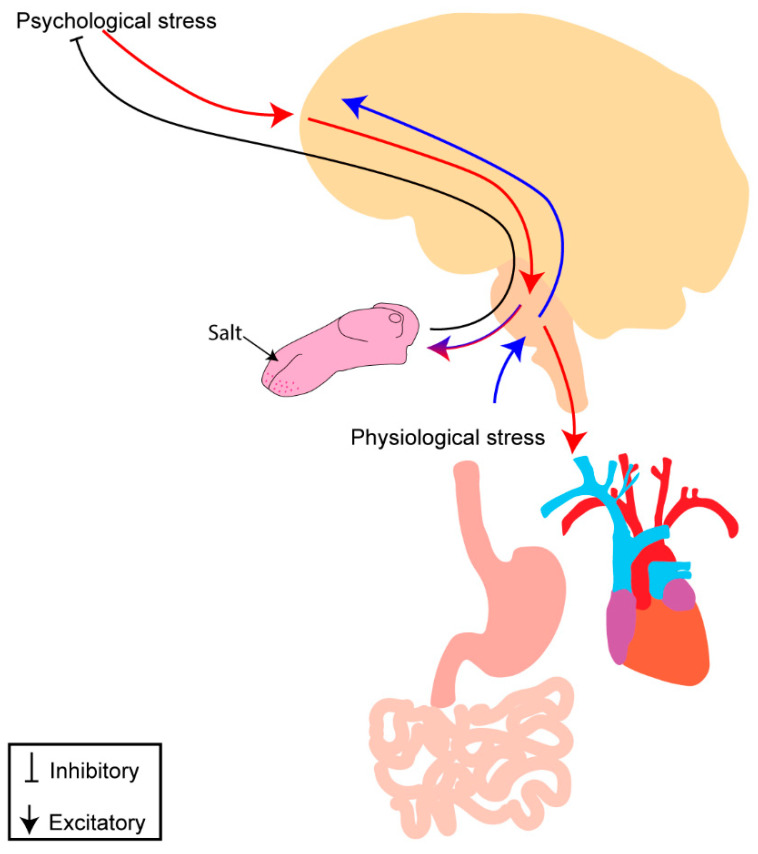
Potential mechanism of interaction between psychological and physiological stress with salt intake. Psychological stress (red) involves top-down processing from cortical regions throughout the brain to areas involved in autonomic control and salt-intake regulation, leading to disruption of visceral organs and development of cardiometabolic disease. Physiological stress (blue) involves bottom–up processing as inputs from the viscera enter the brainstem and are relayed throughout the brain to areas involved in autonomic control, salt intake regulation, and stress responses. Salt intake may be “stress dampening” and result in modulation of psychological and physiological stress processing and alleviate discomfort experienced with a real or perceived threat to homeostasis. Perceived stress (psychological or physiological) may alter gustatory processing, centrally and/or peripherally, to modulate salt taste and increase salt intake.

The link between salt intake and cardiovascular dysfunction is widely acknowledged, particularly in the light of the deleterious and hypertensive consequences of excess sodium ingestion. The inverse relationship—how disease states alter salt intake—is given less credence. In this review, we have highlighted how stress-related and cardiometabolic diseases, specifically obesity and hypertension, alter salt preference resulting in increased salt intake, similar to the shift with sodium appetite observed in other mammalian species subjected to sodium depletion. Our proposition is that stress-related (particularly cardiometabolic) disease states also impact the drive to consume sodium and sensation of sodium taste (Figure 4). The consequence is the potential for a feed-forward mechanism that exacerbates these disease states.

Potential mechanism(s) underlying alterations in salt intake observed in stress-related disease, including CMDs, most likely stem from similarities in neural circuitry and endocrine factors mediating salt intake and blood-pressure regulation. Sensation of changes in [Na^+^] by osmosensitive neurons or changes in blood pressure and volume by baroreceptor afferents initiate neural pathways to regulate salt intake and blood pressure accordingly. Once in the brain, these signals are relayed to many of the same areas (Figure 1) to regulate salt intake and blood pressure. The NTS, PBN, and IC are major sensory hubs that may serve as potential locations for integration of salt taste and intake and cardiovascular information to regulate salt consumption and blood pressure and volume accordingly. The PVN may also contribute to this integration through control of neuroendocrine peptides, such as vasopressin and oxytocin, and ANGII binding. Oxytocin has various roles, including in sodium appetite and cardiovascular control, and central actions of oxytocin inhibit sodium appetite and alter blood pressure. ANGII’s actions on AT1R expressed on neurons within the PVN and throughout the brain alter sodium appetite and cardiovascular responses. Based on the expression of the AT1R and Oxtr throughout gustatory, sodium appetite, and cardiovascular sensory organs and circuitry, it is reasonable to hypothesize that these two peptides may contribute to integration of changes in blood volume, pressure, or osmolality to regulate salt intake accordingly.

Understanding salt intake and cardiovascular sensory integration may provide insight into the mechanism(s) underlying the relationship between salt intake and blood pressure, particularly in the development and maintenance of hypertension. Upon consumption, salt [NaCl] is detected by taste buds on the tongue and gustatory afferents relay this information to the gustatory rNTS. This circuitry overlaps with that of baroreceptor afferents innervating the aortic arch and carotid sinus that transmit this information to the cardiovascular cNTS (Figure 1). ANGII and/or oxytocin may act on their receptors along these afferents to coordinate gustatory and baroreceptor processes to regulate salt intake in response to changes in blood pressure and volume. Future work should investigate this sensory integration in control of salt intake and blood pressure, as it may provide insight into the relationship between salt intake and disease. Enhancing our understanding of interoceptive and gustatory systems as targets for peptides, such as ANGII and oxytocin, may provide the potential of targeting these systems for the prevention or treatment of diseases linked to overconsumption of salt.

## Data Availability

Not applicable.
